# The silicon supplement ‘Monomethylsilanetriol’ is safe and increases the body pool of silicon in healthy Pre-menopausal women

**DOI:** 10.1186/1743-7075-10-37

**Published:** 2013-04-26

**Authors:** Ravin Jugdaohsingh, Maio Hui, Simon HC Anderson, Stephen D Kinrade, Jonathan J Powell

**Affiliations:** 1 MRC Human Nutrition Research, Elsie Widdowson Laboratory, Fulbourn Road, Cambridge, CB1 9NL, UK; 2 Gastrointestinal Laboratory, Rayne Institute, St Thomas’ Hospital, London, UK; 3 Department of Chemistry, Lakehead University, Thunder Bay, ON, Canada

**Keywords:** Oral silicon supplement, Monomethylsilanetriol, Proton Nuclear Magnetic Resonance Spectroscopy, Inductively Coupled Plasma Optical Emission Spectrometry, Orthosilicic acid

## Abstract

**Background:**

Monomethylsilanetriol (MMST) has been used for decades as an oral silicon supplement for bone and connective tissue health, although there are no formal data on its *in vivo* utilisation or safety following sustained dosing.

**Methods:**

To investigate whether MMST contributes to the body pool of silicon and, secondly, to establish its safety following 4 weeks’ supplementation in humans, twenty-two healthy pre-menopausal women (22–38 years) were recruited and supplemented with MMST at the maximum daily recommended dose (10.5 mg Si/day) for 4 weeks in a double-blind, randomised, placebo-controlled, cross-over design (i.e. 8 weeks in total). Fasting serum and urine samples were collected at baseline and at the end of the 4-week supplementation/placebo periods for analysis of total silicon by inductively coupled plasma optical emission spectrometry, MMST by proton nuclear magnetic resonance spectroscopy and full serum biochemistry. Participants also reported on, by questionnaire, their health, well-being and quality of life at 0, 4 and 8 weeks.

**Results:**

Overall, 4-weeks supplementation with MMST significantly increased total fasting Si concentrations in serum and urine (*P* ≤ 0.003; paired *t*-test). MMST was semi-quantifiable in serum and quantifiable in urine, but only accounted for ca. 50% and 10%, respectively, of the increased total-Si concentration. There were no reported adverse effects (i.e. changes to health and well-being) or serum biochemical changes with MMST versus placebo.

**Conclusions:**

Our data indicate that oral MMST is safe, is absorbed and undergoes sufficient metabolism *in vivo* to raise fasting serum silicon levels, consistent with other well absorbed forms of dietary silicon (e.g. orthosilicic acid). It thus appears to be a suitable silicon supplement.

## Background

In spite of much research on dietary silicon in relation to its role in normal bone and connective tissue health, its proposed function [1,2], and especially its essentiality [3,4], remains unproven in higher animals. Indeed, the case for a functional role of dietary silicon in humans (e.g. in optimal connective tissue health) may be best elucidated by a long-term, prospective intervention study but, in which case, a readily bioavailable and safe form of Si must be determined. Monomethylsilanetriol (MMST) has been an over-the-counter Si supplement of long-standing use throughout the world and especially in continental Europe (e.g. France). It does not contain nano-silica particles unlike some silicon supplements and, over which, safety concerns have been expressed [5,6]. However, the European Safety Authority recently advised that insufficient data currently exist to justify the use of MMST as an oral supplement [7].

MMST is a monomeric, organosilicon molecule [Si(OH)_3_CH_3_] that is stable in aqueous solution at high concentrations (~ 20 mM at room temperature; unpublished data) compared to its naturally occurring inorganic analogue (orthosilicic acid, Si(OH)_4_). MMST is readily absorbed following ingestion and no adverse effects have been reported [8-14], although no formal studies on its safety have been undertaken. Moreover, whether cleavage of the Si-CH_3_ bond occurs with bioconversion to the putative bioactive form of Si (i.e. Si(OH)_3_CH_3_ → Si(OH)_3_OH) is not clear. In favour of this hypothesis is that MMST positively influences connective tissues, namely bone [8,11,12] and blood vessels [9] in mammalian studies, in much the same way that dietary Si does [2] and supplementation in rats leads to increased Si levels in the connective tissues (Jugdaohsingh *et al.*, unpublished data). It has been proposed that biological cleavage of the Si-CH_3_ bond in MMST should be possible, based upon clear evidence with other organosilicons *in vivo*[15-19]. The primary objective of this study, therefore, was to gain insight into the metabolism of MMST and its ability, or not, to contribute to the body pool of Si following sustained dosing at the maximum daily recommended dose of this product, in humans.

The ‘body pool’ of Si is currently best assessed in fasting serum samples. Following ingestion, dietary and some supplemental forms of Si are relatively rapidly absorbed into the circulation [14,20-23], and this is especially true for MMST [10,14]. The majority is then rapidly excreted in urine [22-24], but a minority probably undergoes tissue loading and/or cellular metabolism [24] and sustained low dose Si supplementation, with an inorganic source of Si, appears to lead to a marked rise in the body pool of Si, as reflected in fasting serum and urine Si concentrations [25]. Analysis of non-fasting serum levels, however, can lead to falsely high results as the measured Si may include not only that in equilibrium but also that recently absorbed and not yet lost from the circulation. Our previous studies show that serum Si levels fall to baseline 6 hours after ingestion of typical dietary doses of Si, suggesting that this, or later, is the optimum time point for measurement of fasting Si levels [14,20-23]. The actual time frame is both dose- and Si species dependent but for the rapidly absorbed MMST, at typical low doses (e.g. 3.5 mg Si three times per day against a usual dietary background of 20–30 mg per day), post-absorptive clearance is very rapid (a few hours; [10,14]). To be extra careful, in this study, a minimum fasting time between last ingestion and blood or urine sampling of 10 hours was used. A secondary objective of this study was to investigate the safety of MMST supplementation in humans.

## Subjects and methods

### Subjects

**Figure 1:**
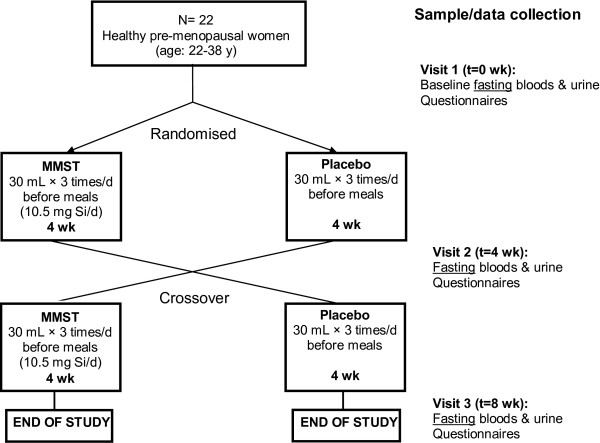
**Study design.** Flow diagram summarising the double-blind, randomised, placebo controlled cross-over study design.

Twenty-two healthy pre-menopausal females, aged between 22 and 38 years, with no history of serious illness and not taking any medication or silicon-containing food supplements, were recruited to the study by circular email from King’s College London (UK) (Table 1). Volunteers were excluded who were pregnant and lactating, of child-bearing age and not taking contraception, or not able to follow the study protocol. Young women were chosen because Si supplementation is likely to be most relevant in this population since epidemiological data have shown a pronounced positive association between dietary Si intakes and bone mineral density in this group [26]. Indeed, it is hypothesised that estradiol is required for Si to have any biological effect [26,27]. The women were randomly assigned to coded Placebo or MMST (silicon supplement: 10.5 mg Si/day) for 4 weeks each, in a crossover design; i.e. 11 participants took placebo for four weeks followed by MMST for four weeks and the remaining 11 participants took MMST for four weeks followed by placebo for four weeks (Figure 1). Anthropometric data (age, weight, height and BMI) were collected for each participant and there were no statistical differences in baseline parameters (see Table 1) or baseline biochemistry (Additional file 1) between the two groups.

**Table 1:** Baseline characteristics of the subjects in the two randomised groups (MMST first followed by placebo or placebo first followed by MMST)

	**MMST first 4 weeks (n=11)**			**Placebo first 4 weeks (n=11)**		
	**Mean**	**sd**	**Range**	**Mean**	**sd**	**Range**
Age (y)	24.6	1.8	22-28	28.5	5.1	23-38
Height (m)	1.65	0.07	1.52-1.73	1.65	0.74	1.60-1.75
Weight (kg)	56.4	4.4	49-62	60.8	6.2	50-69
BMI (kg/m^2^)	20.76	1.12	18.5-22.5	22.44	2.27	19.5-25.6
Serum Creatinine (μmol/L)	66	7	57-78	68	10	51-85

The study was conducted according to the guidelines laid down in the Declaration of Helsinki and was approved by the Research Ethics Committee of King’s College London (UK). All participants gave signed written consent following oral and written explanation of the study details.

### Materials

Ultra-high purity (UHP) water (18 MΩ cm) was from an Elga water purifier (Elga Ltd, High Wycombe, UK) and ^2^H_2_O (99.98%) was from Cambridge Isotopes (Andover, MA, USA). Both were shown by ICP-OES to contain < 5 μg/L Si. Nitric acid (65% (w/v) HNO_3_) was high purity from Fluka Ltd (Gillingham, UK). The needles (21 Gauge) and plastic syringes used for blood collection were from Terumo Europe NV (Leuven, Belgium). Polypropylene transport tubes from Sarstedt Ltd (Leicester, UK) were washed with UHP water and air-dried (in a class J clean room) prior to use. Serology tubes (EDTA, serum separating tube (SST) and fluoride) were from BD (Oxford, UK). Polypropylene Mauser bottles (2.5 L) from Aldrich Chemical Co (Gillingham, UK) were cleaned with 10% (v/v) HNO_3_ (AnalaR; BDH Ltd, UK), thoroughly rinsed with UHP water, air dried and pre-weighed prior to use for urine collection. For ^1^H-NMR analysis of MMST, all labware (HDPE bottles, Teflon FEP syringes, glass NMR tubes) was successively washed in dichloromethane, acetone, aqueous detergent and UHP water so as to eliminate any silicone residue. Pasteur pipettes (3.5 mL) used for sample transfer were from Greiner Bio-One Limited (Stonehouse, UK). Stock silicon ICP standard solution (9,650 mg/L Si) was from Aldrich Chemical Co (Gillingham, UK), whereas pyridine was from Sigma Aldrich Ltd (Oakville, Canada). The MMST silicon supplement and the placebo were prepared, packaged and supplied in a blinded fashion by the manufacturer, namely LLR-G5 Ltd (Castlebar, Ireland).

### Study design

The study was double-blind and of crossover design (Figure 1). All participants were supplemented with MMST (at the maximum daily recommended dose of 10.5 mg Si/d, which is equivalent to 40% of total average daily dietary Si intake in this population [22]) and placebo, each for 4 weeks, but the order was randomised. All data analysis was completed and kept confidential by the authors prior to the codes being sent by the manufacturer.

Participants attended The Gastrointestinal Laboratory at St Thomas’ Hospital on three occasions at four-week intervals. They fasted from 10.30 pm on the night preceding each visit until 9:30 am when their blood and urine samples were collected. Participants were asked to empty their bladder upon waking or, if they had not done so, upon their arrival at The Gastrointestinal Laboratory. Blood and urine (second void) were collected and a quality-of-life questionnaire completed (complete details below). The age, height, weight and body mass index (BMI) of the participants were recorded during the first visit (week-0) and the subjects were randomised to receive 28 coded bottles of either the placebo or MMST supplement, i.e. one bottle per day for 28 days (4 weeks). The two solutions (MMST and placebo) were identical in appearance, colour, taste, odour and packaging. Participants were instructed to take 30 mL of either MMST (3.5 mg Si) or placebo three times a day before meals (breakfast, lunch and dinner) during the next four weeks. To aid with compliance, a dosing cup was provided to each participant and all solutions were contained in 90 mL plastic bottles. At the second visit (week-4), participants were given 28 bottles of the opposite solution to that given at visit-1 and instructed to maintain the same dosing regimen until returning for the next visit (week-8).

### Blood and urine collections

Twenty mL of blood was collected from a forearm vein of each fasted participant, and transferred for later analysis to: a) an EDTA tube; b) a fluoride (glucose) tube; c) two SST (biochemistry) tubes; and d) a Si- and anticoagulant-free polypropylene tube. The latter sample was left to clot at room temperature for one hour before being centrifuged at 2,000 *g* and 4°C for 10 min; the separated serum was then transferred to a second Si-free polypropylene tube and stored at −20°C for later determination of total-Si and MMST content.

Second-void urine was also collected. Prior to each visit, participants emptied their bladder upon waking (first-void urine) and then only consumed, *ad libitum*, UHP water up to the time they collected their second-void urine (between 08.30-09.30 a.m.) in a polypropylene Mauser bottle. Ten mL of the fresh, homogenous urine was then transferred into a 25 mL polypropylene bottle and diluted with 10 mL nitric acid (0.7%, ultra-high-purity) to prevent precipitation of minerals during storage at 4°C. A subset of these urine samples was analysed for MMST as detailed below.

### Analyses

#### Total-silicon

Total-Si analysis was carried out by inductively coupled plasma optical emission spectrometry (Jobin-Yvon JY24; Instruments SA, Lonjumeau, France) with a V-groove nebuliser and a Scott-type double-pass spray chamber at 251.611 nm. Sample flow rate was 1 mL/min and peak profiles were assessed as before [20,28]. Standards and samples were analysed at least in duplicate.

Serum samples were thawed at room temperature and diluted four-fold with dilute high-purity nitric acid; i.e. 3 mL of 0.26% (v/v) nitric acid was added to 1 mL serum. Sample-based standards were prepared by pooling 1 mL of each diluted serum sample and then spiking aliquots with varying amounts of Si (0, 50, 100 or 200 μg/L), using a working stock silicon solution of 101.60 mg/L that was prepared from silicon ICP standard. The sample ‘blank’ was 0.26% (v/v) nitric acid.

Urine samples (diluted 1:1 with 0.7% (v/v) nitric acid) were incubated overnight in an oven at 40°C prior to analysis, to re-solubilise any precipitates formed during storage. One mL of each diluted urine sample was pooled and, again, aliquots of this pooled sample were spiked with 0, 5, 10 or 20 mg/L Si to make the ‘pooled sample-based standards’.

### MMST by ^1^H-NMR

A subset of the fasting serum (n=9) and urine samples (n=10) following MMST supplementation were analysed by ^1^H-NMR, firstly to determine whether MMST could be detected and secondly to investigate whether it was converted/metabolised to OSA. Samples were chosen based on the likelihood of detecting MMST, that is, those which exhibited large increases in total-Si concentration compared to baseline. Baseline serum (n=3) and urine (n=5) samples which contained high total-Si were used as controls, along with placebo samples (n=4 serum and urine) which exhibited apparent increases in total-Si compared to baseline.

Approximately 0.6 g sample and 0.2 g ^2^H_2_O (containing 0.025 wt% pyridine as an internal quantitative nuclear magnetic resonance (QNMR) reference) were precisely weighed into 5 mm O.D. NMR tubes. A series of QNMR calibration standards were similarly prepared using dilute stock solutions of MMST (Dow Corning 777) and potassium hydrogen phthalate (Sigma Aldrich). One-dimensional solvent-supressed ^1^H NMR spectra were acquired at 25°C on a Varian Unity Inova 500 spectrometer, using 90° observe pulses (375 for urine, 700 for serum) and a 4 s recycle period.

### Blood biochemistry

The safety of MMST was assessed by changes in serum biochemistry following supplementation with MMST and placebo. Fasting blood samples collected at all three time points were sent to the Clinical Biochemistry Laboratory at St Thomas' Hospital for clinical assessment of renal function, liver function, lipid profile, thyroid stimulating hormones (TSH), C reactive protein (CRP) and other blood analytes to determine the safety of the MMST and placebo solutions. Samples were analysed on a clinical chemistry analyser (Roche Modular Analytics (Roche Diagnostics Ltd, Switzerland), P module for routine chemistry and E module for hormones/tumour markers etc.).

### Health, wellness and quality of life

As a further check on the safety of the MMST and placebo solutions, at each visit the participants completed a questionnaire in which they self-assessed their well-being and quality of life. The questionnaires were created specifically for this study (see Additional file 1) but were adapted from typical such questionnaires in the art (see: http://www.donaldepstein.com/pdf/newlongitudinal.pdf for example).

### Statistical analysis

Results are expressed as means ± 1 standard deviation (SD), unless otherwise stated. Of the 22 recruited participants, complete serum datasets (total-Si concentrations) were only available for 14 participants (i.e. data for all three collections: week-0 (“baseline”), week-4 and week-8), allowing paired analyses, while 18 participants had complete urine datasets. Of the eight participants with incomplete serum dataset: one did not have values for all three time points (but did have urine data); five had no baseline values; one subject had no values at baseline and week 4, while another participant had no value at week 4. Reasons were: unable to collect or provide a blood sample, Si concentration in the diluted baseline serum sample for analysis was below the limit of quantification (44 μg/L), or subject did not fast. All subjects had quantifiable fasting serum Si concentration after 4 weeks supplementation with MMST. Of the four subjects with incomplete urine dataset: two subjects had no values for all three time points; one subject did not have a value for week 4; and one subject did not have a value for week 8. Reasons were: unable to provide a sample, urine infection, or subject did not fast. Differences between groups were analysed with a paired (samples) *t*-test. *P*<0.05 was considered to be significant. To correct for multiplicity of testing, a Bonferroni Correction was applied to the *P* value. No previous human data exist for sample size (power) calculation.

## Results

### Metabolism of MMST

#### Fasting total serum silicon

**Figure 2:**
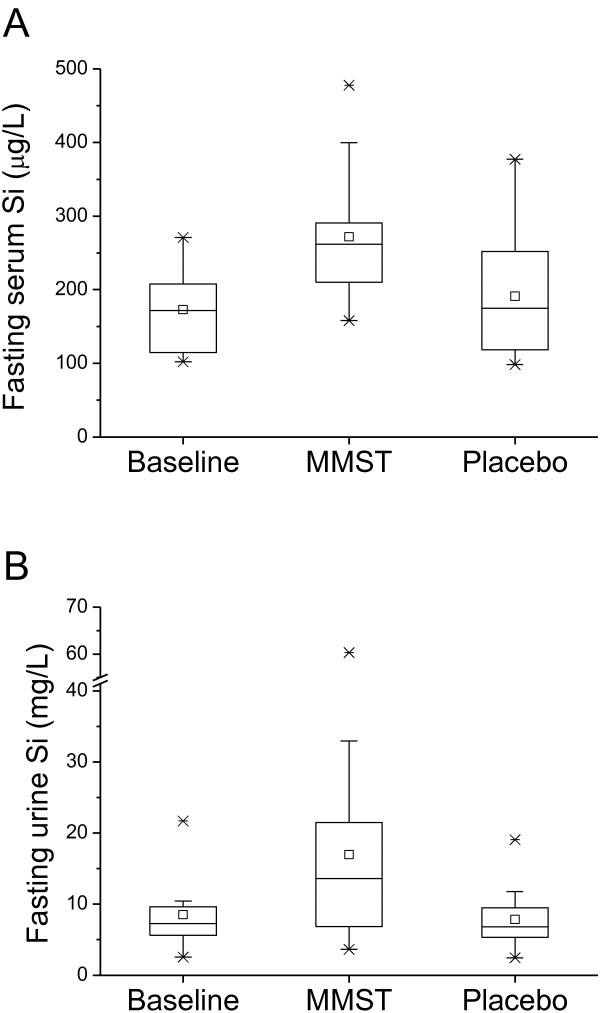
**Fasting serum and urinary silicon levels before and after supplementation with MMST and placebo.** (**A**) Fasting serum total-Si concentrations before supplementation (Baseline: n = 14) or following 4 weeks supplementation with organic silicon (MMST: n = 14) or 4 weeks of placebo (Placebo: n =14). Data is shown as box-plots, where the horizontal lines indicate the 5^th^, 25^th^, 50^th^ (or median), 75^th^ and 95^th^ percentiles, the open square shows the mean and the crosses the minimum and maximum values. Total-Si concentrations were significantly higher following supplementation with MMST compared to Baseline (*P* = 0.0002; paired *t*-test) or Placebo (*P* = 0.003; paired *t*-test). No effect of the order in receiving the solutions (i.e. MMST before or after placebo) was observed. (**B**) Fasting urine total-Si concentrations before supplementation (Baseline: n = 18) or following 4 weeks supplementation with organic silicon (MMST: n = 18) or 4 weeks of placebo (Placebo: n =18). Total-Si concentration was significantly higher following supplementation with MMST compared to Baseline (*P* = 0.008; paired *t*-test) or Placebo (*P* = 0.007; paired *t*-test). No effect of the order in receiving the solutions (i.e. MMST before or after placebo) was observed.

For the 14 participants with complete serum datasets, four weeks of supplementation with MMST led to a markedly significant increase in fasting serum silicon concentration (mean 272 μg/L) compared to baseline (mean 173 μg/L: *P* = 0.0002) or to placebo (mean 191 μg/L: *P* = 0.003) (Figure 2A). There was no effect of treatment order (i.e. MMST before placebo or placebo before MMST; data not shown).

### Fasting total urinary silicon

For the 18 participants with complete urinary datasets, four weeks of supplementation with MMST led to a marked increase in fasting urinary silicon concentrations (mean 17.0 mg/L) compared to baseline (mean 8.5 mg/L: *P* = 0.008) and placebo (mean 7.8 mg/L: *P* = 0.007) (Figure 2B). There was no effect of order (i.e. MMST before placebo or placebo before MMST; data not shown).

**Figure 3:**
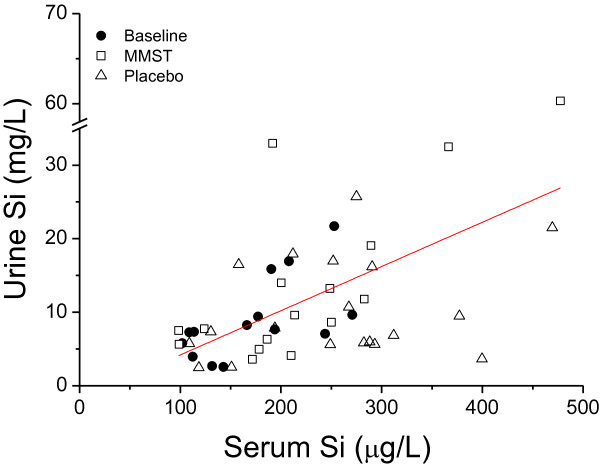
**Correlation between fasting serum and urinary silicon levels.** Correlation (r = 0.55 and *P* < 0.0001; n = 49) between individual fasting urine total-Si concentrations and the corresponding (i.e. paired) fasting serum total-Si concentrations at baseline (*solid circles*) and after supplementation with MMST (*open squares*) and placebo (*open triangles*).

As previously observed for dietary silicon, fasting urinary silicon concentrations correlated closely with fasting serum silicon concentrations throughout the study period (Figure 3).

### Silicon as MMST in the urine and serum samples

**Figure 4:**
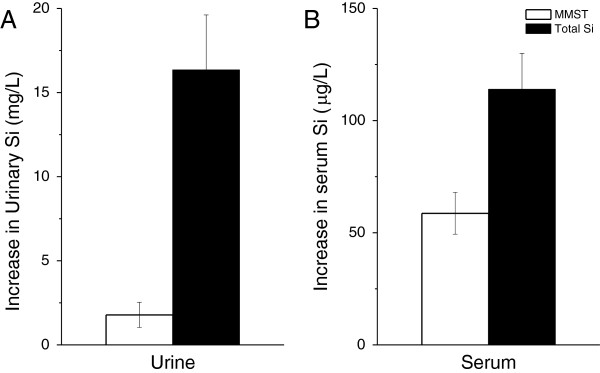
**Increase in fasting serum and urinary silicon levels detected as MMST.** Increase of total-Si concentration (*black bars*) and MMST concentration (*white bars*) in fasting urine (**A**, n=10) and fasting serum (**B**, n=6) following 4-week supplementation with MMST. MMST was not detected in the baseline serum samples (detection limit ca. 10 μg/L), but was detected in three of the baseline urine samples (21 ± 7 μg/L; detection limit 3 μg/L).

We used ^1^H-NMR to quantitate the concentration of MMST in urine and compared that value to the total-Si increase (as determined by ICP-OES) over and above baseline in the paired samples. MMST was detected above the detection limit (3 μg/L) in all 10 fasting urine samples following supplementation with MMST (Figure 4A). Curiously, MMST was also detected, albeit at much lower concentrations, in three of the five baseline samples (week-0) and three of the four placebo samples (week-4) analysed (data not shown). The concentration of MMST in the 10 fasting urine samples following MMST supplementation accounted for only 10.3 ± 6.6% of the increase in the total-Si excreted, consistent with significant metabolism of the organosilicon to inorganic silicon.

The serum analysis was hampered by the limits of detection of ^1^H-NMR in this sample type (ca. 10 μg/L). Nonetheless, in 6/9 samples, semi-quantitation could be achieved and it was consistent with significant metabolism of MMST: the concentration of Si detected as MMST in the six fasting serum samples accounted for 50.6 ± 13.5% of the increase in total-Si (Figure 4B). MMST was present below the detection limit in the baseline (n = 3) and placebo (n = 4) samples analysed (data not shown).

### Safety of MMST

#### Serum biochemistry

There were no changes in serum biochemistry following Si supplementation, and no effect of the sequence of supplementation (i.e. MMST followed by placebo or placebo followed by MMST), to markers of renal function or liver function, lipid profile, inflammation, or other blood analytes (Additional file 1).

### Health, wellbeing and quality of life

There were no changes in the participants’ health, well-being and quality of life following supplementation with MMST or placebo (Additional file 1).

## Discussion

Our data provide evidence that, following ingestion, MMST [Si(OH)_3_CH_3_] is converted to orthosilicic acid [Si(OH)_4_] and that supplemental use of MMST is safe.

Following four weeks of continuous supplementation with MMST (at the maximum recommended dose of 3.5 mg Si three times per day), fasting serum and urine Si concentrations were, on average, doubled. There appear to be two routes of bio-distribution for dietary silicon following its absorption: namely, rapid urinary excretion for the majority [22-24] and tissue loading and/or cellular metabolism for a minority [24]. As a result, sustained low dose silicon supplementation leads to a marked rise in the body pool of silicon [25], presumably as the second, minor pathway is increasingly loaded. The biologically important target sites for dietary and supplemental silicon appear to be the connective tissues, such as blood vessels [9,29], joints, skin [30-32] and, especially, bone [2,8,11,12,26,33]. Thus the increase in fasting serum and urine Si concentrations following MMST supplementation must result from silicon that has entered the metabolic pool and not that which is destined for immediate urinary excretion following absorption. We were careful to avoid the latter by using fasting serum and urine samples following a minimum of a 10 h fast and previous data has shown this to be more than adequate [10,14,24]. This marked increase in the body pool of silicon supports the argument that MMST is an effective silicon supplement and also explains why this form of silicon has been shown to positively influence bone and blood vessels in mammalian studies [8,9,11,12] in much the same way that dietary silicon does [1,26,27,30-33].

Fasting serum Si levels in this study, before and after supplementation with MMST, were variable, but baseline levels were comparable to what we have previously observed for pre-menopausal women [34]. A few baseline samples were below the LOQ, but this is not unusual for this population and probably reflects either the ethnic diversity of the group with their different dietary Si exposure [2,35] and/or differences in circulating hormonal levels that could, in theory, impact upon serum and tissue Si levels, although further work is required to confirm this [34,36]. Even with this variable Si baseline, MMST supplementation significantly increased fasting serum Si level above baseline. Whether this increase in serum Si level is beneficial is not known and was not the aim of this study. This would require a different study design with, probably, a longer period of supplementation. Conversely, the fact that fasting serum Si levels can be increased with Si supplementation could be interpreted to mean that normal dietary Si intake/exposure is sub-optimal but, again, the absence of any known Si deficiency state or symptoms makes this impossible to investigate directly. Nonetheless, as mentioned above, increased dietary Si intake is associated with increased bone mineral density at least in men and pre-menopausal women [26,27].

Importantly, we provide evidence for the conversion/metabolism of MMST to OSA; namely that organosilicon concentration in urine and serum accounted for only 10% and ca. 50% of the increase in total-Si in the fasting urine and serum samples, respectively. No previous data exist, specifically, for the conversion or metabolism of MMST to OSA. There are, however, data showing that methyl silanes per se (Si-CH_3_) are broken down/converted to silanols (Si-OH) in both the acid environment of the stomach and systemically [15,17-19].

In designing the study, we considered how bioconversion of this proposed supplemental silicon [Si(OH)_3_CH_3_] to OSA [Si(OH)_4_] could be demonstrated in humans. Stable isotopic labelling, with ^29^Si, was discounted due to its relative high natural abundance (ca. 5% of all endogenous Si(OH)_4_) and the relative insensitivity of ^29^Si by NMR. Experiments with ^32^Si would not, these days, allow for administration to humans due to radioactive exposure and long half-life (170 years). Thus, we rationalised that if Si loading (i.e. increased fasting serum levels) is observed following sub-chronic ingestion of MMST in volunteers, as observed for absorbable dietary/supplemental silicon [1,25], then quantitation for MMST (by ^1^H-NMR) together with quantitative ICP-OES for the increase in total silicon, would allow for bio-conversion to be proven by ‘balance’. Moreover, ^1^H-NMR would have detected the conversion of MMST to other organosilicons, although MMST is the smallest organosilicon molecule (building block) and its *in vivo* anabolism to form larger organosilicon species was neither anticipated nor seen.

Interestingly, and surprisingly, MMST was also detected in baseline urine samples and it is likely that this is due to exposure to other organosilicon compounds that are metabolised through this pathway (i.e. via MMST to OSA). Notably, silicones (e.g. dimethylpolysiloxane, E900) are added to foods such as juice and beer as an anti-foaming agent [7,37].

Finally, the serum biochemistry and quality of life data over the 4-week periods during which participants received MMST would suggest that this form of silicon is safe upon sustained dosing at supplemental levels (10.5 mg/day or less). This finding is in accordance with reported studies of MMST being administered orally and intravenously in humans [8,9,11] and with its long-standing use as a supplement without reported adverse events. Data in rats with the same and higher doses of MMST, over a longer (90 day) supplementation period, have also confirmed the safety of this material (unpublished data).

## Conclusions

In conclusion, our data provide evidence for the metabolism and safety of MMST at supplemental levels. The marked increase in the body pool of silicon and evidence of its conversion/metabolism to OSA supports the argument that MMST is an effective silicon supplement. Intervention trials are now warranted.

## Abbreviations

Si: Silicon; MMST: Monomethylsilanetriol; H-NMR: Proton Nuclear Magnetic Resonance; ICP-OES: Inductively coupled plasma optical emission spectrometry; OSA: Orthosilicic acid.

## Competing interests

The authors’ laboratories have received research funding from the silicon supplement and food industry; for the work presented here the MMST and placebo solutions and costs of ^1^H-NMR analysis were provided by LLR-G5 Ltd (Castlebar, Ireland). The research was designed, executed, analysed and communicated only by the authors. JJP and SDK have consulted to LLR-G5 Ltd and others in the silicon supplement industry. All other authors have no competing interest.

## Authors’ contributions

The authors’ contributions were as follows: RJ and JJP designed the research; RJ, MH, SA and SK conducted the research; RJ and MA had study oversight; RJ, MH and SK analysed the data; RJ and JJP wrote the paper & had primary responsibility for final content. All authors read and approved the final manuscript.

## Supplementary Material

Additional file 1 Supplementary data supporting the results of this article is included in an additional file. Click here for file
