# Supplementary material for: The silicon supplement ‘Monomethylsilanetriol’ is safe and increases the body pool of silicon in healthy Pre-menopausal women
Source: Nutr Metab (Lond). 2013 Apr 26;10:37. doi: 10.1186/1743-7075-10-37 (PMC3649945; doi:10.1186/1743-7075-10-37)
Supplement: Additional file 1 — Supplementary data supporting the results of this article is included in an additional file. [file 1743-7075-10-37-S1.docx]

**Supplemental Table.**

Full clinical serum biochemistry data for all subjects before supplementation (Baseline) or, in a cross-over design, following 4 weeks of supplementation with organic silicon (MMST) or 4 weeks of placebo (Placebo)*^1^*

| Test | Baseline  (n=21) | 4 weeks of MMST  (n=21) | 4 weeks of  Placebo  (n=21) | *p-*value*^8^* |
| --- | --- | --- | --- | --- |
| Urea (mmol/L) | 4.29 (0.93) | 4.23 (0.97) | 4.20 (1.0) | 0.893 |
| Creatinine (µmol/L) | 66.0 (8.5) | 67.1 (9.0) | 68.2 (10.8) | 0.239 |
| Calcium (mmol/L) | 2.27 (0.08) | 2.30 (0.09) | 2.29 (0.08) | 0.589 |
| Phosphate (mmol/L) | 1.25 (0.23) | 1.33 (0.16) | 1.30 (0.16) | 0.549 |
| ALP (IU/L)*^2^* | 48.0 (12.3) | 47.8 (11.8) | 47.7 (12.3) | 0.924 |
| Bilirubin (µmol/L) | 10.0 (3.6) | 12.4 (6.6) | 11.5 (4.8) | 0.493 |
| γ GT (U/L)*^3^* | 11.8 (3.1) | 12.9 (5.8) | 12.9 (4.4) | 1.000 |
| Cholesterol (mmol/L) | 4.50 (0.88) | 4.56 (0.84) | 4.49 (0.76) | 0.603 |
| Triglycerides (mmol/L) | 0.846 (0.287) | 0.842 (0.329) | 0.883 (0.310) | 0.605 |
| TSH (mIU/L)*^4^* | 2.58 (1.06) | 2.08 (0.66) | 2.85 (1.21) | 0.158 |
| Glucose (mmol/L) | 4.56 (0.32) | 4.40 (0.44) | 4.43 (0.34) | 0.884 |
| Albumin (g/L) | 46.1 (2.4) | 46.8 (2.8) | 46.8 (1.9) | 0.918 |
| Total Protein (g/L) | 74.7 (4.5) | 75.5 (4.3) | 76.1 (4.8) | 0.518 |
| GFR (mL/min)*^5^* | 95.7 (14.4) | 93.5 (13.4) | 90.6 (16.9) | 0.240 |
| Anion Gap (mmol/L) | 18.3 (2.5) | 18.0 (2.6) | 17.7 (3.1) | 0.688 |
| ALT (IU/L)*^6^* | 18.5 (16.7) | 19.4 (21.7) | 17.0 (11.3) | 0.331 |
| CRP (mg/L)*^7^* | < 5.0 | < 5.0 | < 5.0 |  |
| Magnesium (mmol/L) | 0.816 (0.061) | 0.873 (0.086) | 0.848 (0.055) | 0.478 |
| Potassium (mmol/L) | 4.41 (0.25) | 4.32 (0.26) | 4.42 (0.31) | 0.230 |
| Sodium (mmol/L) | 142 (3) | 141 (3) | 141 (3) | 0.852 |
| Bicarbonate (mmol/L)  Chloride (mmol/L) | 24.2 (1.8)  105 (2) | 24.2 (2.3)  103 (2) | 24.4 (2.0)  103 (2) | 0.662  0.835 |

*^1^*Mean (SD)

*^2^*ALP = alkaline phosphatase. *^3^*γGT = Gamma glutamyl transferase. *^4^*TSH = thyroid stimulating hormone. *^5^*GFR = estimated glomerular filtration rate. *^6^*ALT = alanine aminotransferase. *^7^*CRP = C-reactive protein.

*^8^*Placebo *vs*. MMST. Significance was assessed by paired t-tests. No effect of order (i.e. MMST before or after placebo) was observed. In all cases there were no significant differences between the three periods.

**Supplemental Figure.**

Volunteers’ self-assessment of health, well-being and quality of life (using questionnaire below), following 4 weeks supplementation with MMST (*hollow circles*) or Placebo (*solid circles*). A score > 3 (or > 2 for overall impression) indicated a worsening from their typical state, a score of < 3 (or < 2 for overall impression) indicated an improvement, and a score of 3 (or 2 for overall impression) indicated no change from their typical state. There was no marked change/deviation from the typical state following MMST supplementation or between treatments (MMST versus Placebo). Data are mean ± SD of 21 subjects.

**Supplemental Figure**

**Health, Wellness & Quality of Life Questionnaire (Baseline, Visit 1)**

**Subject code:**……………................... **Date:**……………………………….

Answer each of the questions below by putting a circle around the number that best represents you at this time.

| **I. Physical State** | | | | | | |
| --- | --- | --- | --- | --- | --- | --- |
| **Rate the following questions with respect to frequency:** | | **Never** | **Rarely** | **Occasionally** | **Regularly** | **Constantly** |
| 1. | Presence of physical pain (neck/back ache, sore arms/legs, etc.). | 1 | 2 | 3 | 4 | 5 |
| 2. | Feeling of tension or stiffness or lack of flexibility in your spine. | 1 | 2 | 3 | 4 | 5 |
| 3. | Incidence of fatigue or low energy. | 1 | 2 | 3 | 4 | 5 |
| 4. | Incidence of headaches (of any kind). | 1 | 2 | 3 | 4 | 5 |
| 5. | Incidence of heartburn/chest pain | 1 | 2 | 3 | 4 | 5 |
| 6. | Incidence of nausea or constipation. | 1 | 2 | 3 | 4 | 5 |
| 7. | Incidence of other digestive complaints | 1 | 2 | 3 | 4 | 5 |
| 8. | Incidence of menstrual discomfort. | 1 | 2 | 3 | 4 | 5 |
| 9. | Incidence of allergies or skin rashes. | 1 | 2 | 3 | 4 | 5 |
| 10. | Incidence of dizziness or light-headedness. | 1 | 2 | 3 | 4 | 5 |
| 11. | Incidence of accidents or near accidents or falling or tripping. | 1 | 2 | 3 | 4 | 5 |
| 12. | Incidence of poor quality sleep | 1 | 2 | 3 | 4 | 5 |
| Specific comments: | | | | | | |

| **II. Mental/Emotional State** | | | | | | | | | | | |
| --- | --- | --- | --- | --- | --- | --- | --- | --- | --- | --- | --- |
| **Rate the following questions with respect to frequency:** | | | **Never** | | **Rarely** | | **Occasionally** | | **Regularly** | | **Constantly** |
| 1. | If pain is present, how distressed are you about it? | | 1 | | 2 | | 3 | | 4 | | 5 |
| 2. | Presence of negative or critical feelings about your self. | | 1 | | 2 | | 3 | | 4 | | 5 |
| 3. | Experience of moodiness. | | 1 | | 2 | | 3 | | 4 | | 5 |
| 4. | Experience of depression or lack of interest. | | 1 | | 2 | | 3 | | 4 | | 5 |
| 5. | Being overly worried about small things. | | 1 | | 2 | | 3 | | 4 | | 5 |
| 6. | Difficulty thinking or concentrating or indecisiveness. | | 1 | | 2 | | 3 | | 4 | | 5 |
| 7. | Experience of vague fears or anxiety. | | 1 | | 2 | | 3 | | 4 | | 5 |
| 8. | Being fidgety or restless; difficulty sitting still. | | 1 | | 2 | | 3 | | 4 | | 5 |
| 9. | Difficulty falling or staying asleep. | | 1 | | 2 | | 3 | | 4 | | 5 |
| 10. | Experience of recurring thoughts or dreams. | | 1 | | 2 | | 3 | | 4 | | 5 |
| Specific comments:  **III. Stress Evaluation** | | | | | | | | | | | |
| **Evaluate your stress relative to the following:** | | **None** | | **Slight** | | **Moderate** | | **Pronounced** | | **Extensive** | |
| 1. | Family. | 1 | | 2 | | 3 | | 4 | | 5 | |
| 2. | Significant Relationship. | 1 | | 2 | | 3 | | 4 | | 5 | |
| 3. | Health. | 1 | | 2 | | 3 | | 4 | | 5 | |
| 4. | Finances. | 1 | | 2 | | 3 | | 4 | | 5 | |
| 5. | Work/college. | 1 | | 2 | | 3 | | 4 | | 5 | |
| 8. | General well-being. | 1 | | 2 | | 3 | | 4 | | 5 | |
| 6. | Emotional well-being. | 1 | | 2 | | 3 | | 4 | | 5 | |
| 7. | Coping with daily problems. | 1 | | 2 | | 3 | | 4 | | 5 | |
| Specific comments: | | | | | | | | | | | |

| **IV. Life Enjoyment** | | | | | | |
| --- | --- | --- | --- | --- | --- | --- |
| **Rate the following on a degree scale of 1-5:** | | **Not at all** | **Slight** | **Moderate** | **Considerable** | **Extensive** |
| 1. | Openness to guidance to your "inner voice/feelings." | 1 | 2 | 3 | 4 | 5 |
| 2. | Experience of relaxation or ease or well-being. | 1 | 2 | 3 | 4 | 5 |
| 3. | Presence of positive feelings about yourself. | 1 | 2 | 3 | 4 | 5 |
| 4. | Interest in maintaining a healthy lifestyle (e.g., diet, fitness, etc). | 1 | 2 | 3 | 4 | 5 |
| 5. | Feeling of being open and aware/connected when relating to others. | 1 | 2 | 3 | 4 | 5 |
| 6. | Level of confidence in your ability to deal with adversity. | 1 | 2 | 3 | 4 | 5 |
| 7. | Level of compassion for, and acceptance of, others. | 1 | 2 | 3 | 4 | 5 |
| 8. | Satisfaction with the level of recreation in your life. | 1 | 2 | 3 | 4 | 5 |
| 9. | Incidence of feelings of joy or happiness. | 1 | 2 | 3 | 4 | 5 |
| 10. | Time devoted to things you enjoy. | 1 | 2 | 3 | 4 | 5 |
| Specific comments: | | | | | | |

| **V. Overall Quality of Life** | | | | | | | | |
| --- | --- | --- | --- | --- | --- | --- | --- | --- |
| **Evaluate your feelings relative to the quality of life:** | | **Terrible** | **Unhappy** | **Mostly Dissatisfied** | **Mixed** | **Mostly Satisfied** | **Pleased** | **Delighted** |
| 1. | Your personal life. | 1 | 2 | 3 | 4 | 5 | 6 | 7 |
| 2. | Your romantic life. | 1 | 2 | 3 | 4 | 5 | 6 | 7 |
| 3. | Your job/education. | 1 | 2 | 3 | 4 | 5 | 6 | 7 |
| 4. | The actual work you do. | 1 | 2 | 3 | 4 | 5 | 6 | 7 |
| 5. | The handling of problems in your life. | 1 | 2 | 3 | 4 | 5 | 6 | 7 |
| 6. | What you are actually accomplishing in your life. | 1 | 2 | 3 | 4 | 5 | 6 | 7 |
| 7. | Your physical appearance - the way you look to others. | 1 | 2 | 3 | 4 | 5 | 6 | 7 |
| 8. | Your ability to adjust to change in your life. | 1 | 2 | 3 | 4 | 5 | 6 | 7 |
| 9. | Your life as a whole. | 1 | 2 | 3 | 4 | 5 | 6 | 7 |
| 10. | Overall contentment with your life. | 1 | 2 | 3 | 4 | 5 | 6 | 7 |
| 11. | The extent to which your life has been as you want it. | 1 | 2 | 3 | 4 | 5 | 6 | 7 |
| Specific comments: | | | | | | | | |

**Health, Wellness & Quality of Life Questionnaire (4 & 8 weeks)**

**Subject code:**……………................... **Date:**……………………………….

Answer each of the questions below by putting a circle around the number that best represents you at this time.

| **I. Physical State** | | | | | | |
| --- | --- | --- | --- | --- | --- | --- |
| **Please assess how each of the following have changed over the past 4 weeks compared to your typical state:** | | **Much better** | **Better** | **The same** | **Worse** | **Much worse** |
| 1. | Presence of physical pain (neck/back ache, sore arms/legs, etc.). | 1 | 2 | 3 | 4 | 5 |
| 2. | Feeling of tension or stiffness or lack of flexibility in your spine. | 1 | 2 | 3 | 4 | 5 |
| 3. | Incidence of fatigue or low energy. | 1 | 2 | 3 | 4 | 5 |
| 4. | Incidence of headaches (of any kind). | 1 | 2 | 3 | 4 | 5 |
| 5. | Incidence of heartburn/chest pain | 1 | 2 | 3 | 4 | 5 |
| 6. | Incidence of nausea or constipation. | 1 | 2 | 3 | 4 | 5 |
| 7. | Incidence of other digestive complaints | 1 | 2 | 3 | 4 | 5 |
| 8. | Incidence of menstrual discomfort. | 1 | 2 | 3 | 4 | 5 |
| 9. | Incidence of allergies or skin rashes. | 1 | 2 | 3 | 4 | 5 |
| 10. | Incidence of dizziness or light-headedness. | 1 | 2 | 3 | 4 | 5 |
| 11. | Incidence of accidents or near accidents or falling or tripping. | 1 | 2 | 3 | 4 | 5 |
| 12. | Incidence of poor quality sleep | 1 | 2 | 3 | 4 | 5 |
| Specific comments: | | | | | | |

| **II. Mental/Emotional State** | | | | | | | | |
| --- | --- | --- | --- | --- | --- | --- | --- | --- |
| **Please assess how each of the following have changed over the past 4 weeks compared to your typical state:** | | **Much better** | | **Better** | **The same** | | **Worse** | **Much worse** |
| 1. | If pain is present, how distressed are you about it? | 1 | | 2 | 3 | | 4 | 5 |
| 2. | Presence of negative or critical feelings about your self. | 1 | | 2 | 3 | | 4 | 5 |
| 3. | Experience of moodiness. | 1 | | 2 | 3 | | 4 | 5 |
| 4. | Experience of depression or lack of interest. | 1 | | 2 | 3 | | 4 | 5 |
| 5. | Being overly worried about small things. | 1 | | 2 | 3 | | 4 | 5 |
| 6. | Difficulty thinking or concentrating or indecisiveness. | 1 | | 2 | 3 | | 4 | 5 |
| 7. | Experience of vague fears or anxiety. | 1 | | 2 | 3 | | 4 | 5 |
| 8. | Being fidgety or restless; difficulty sitting still. | 1 | | 2 | 3 | | 4 | 5 |
| 9. | Difficulty falling or staying asleep. | 1 | | 2 | 3 | | 4 | 5 |
| 10. | Experience of recurring thoughts or dreams. | 1 | | 2 | 3 | | 4 | 5 |
| Specific comments:  **III. Stress Evaluation** | | | | | | | | |
| **Please assess how each of the following stresses have changed over the past 4 weeks compared to your typical state:** | | | **Much better** | **Better** | | **The same** | **Worse** | **Much worse** |
| 1. | Family. | | 1 | 2 | | 3 | 4 | 5 |
| 2. | Significant Relationship. | | 1 | 2 | | 3 | 4 | 5 |
| 3. | Health. | | 1 | 2 | | 3 | 4 | 5 |
| 4. | Finances. | | 1 | 2 | | 3 | 4 | 5 |
| 5. | Work/college. | | 1 | 2 | | 3 | 4 | 5 |
| 8. | General well-being. | | 1 | 2 | | 3 | 4 | 5 |
| 6. | Emotional well-being. | | 1 | 2 | | 3 | 4 | 5 |
| 7. | Coping with daily problems. | | 1 | 2 | | 3 | 4 | 5 |
| Specific comments: | | | | | | | | |

| **IV. Life Enjoyment** | | | | | | |
| --- | --- | --- | --- | --- | --- | --- |
| **Please assess how each of the following have changed over the past 4 weeks compared to your typical state:** | | **Much better** | **Better** | **The same** | **Worse** | **Much worse** |
| 1. | Openness to guidance to your "inner voice/feelings." | 1 | 2 | 3 | 4 | 5 |
| 2. | Experience of relaxation or ease or well-being. | 1 | 2 | 3 | 4 | 5 |
| 3. | Presence of positive feelings about yourself. | 1 | 2 | 3 | 4 | 5 |
| 4. | Interest in maintaining a healthy lifestyle (e.g., diet, fitness, etc). | 1 | 2 | 3 | 4 | 5 |
| 5. | Feeling of being open and aware/connected when relating to others. | 1 | 2 | 3 | 4 | 5 |
| 6. | Level of confidence in your ability to deal with adversity. | 1 | 2 | 3 | 4 | 5 |
| 7. | Level of compassion for, and acceptance of, others. | 1 | 2 | 3 | 4 | 5 |
| 8. | Satisfaction with the level of recreation in your life. | 1 | 2 | 3 | 4 | 5 |
| 9. | Incidence of feelings of joy or happiness. | 1 | 2 | 3 | 4 | 5 |
| 10. | Time devoted to things you enjoy. | 1 | 2 | 3 | 4 | 5 |
| Specific comments: | | | | | | |

| **V. Overall Quality of Life** | | | | | | |
| --- | --- | --- | --- | --- | --- | --- |
| **Please assess how each of the following have changed over the past 4 weeks compared to your typical state:** | | **Much better** | **Better** | **The same** | **Worse** | **Much worse** |
| 1. | Your personal life. | 1 | 2 | 3 | 4 | 5 |
| 2. | Your romantic life. | 1 | 2 | 3 | 4 | 5 |
| 3. | Your job/education. | 1 | 2 | 3 | 4 | 5 |
| 4. | The actual work you do. | 1 | 2 | 3 | 4 | 5 |
| 5. | The handling of problems in your life. | 1 | 2 | 3 | 4 | 5 |
| 6. | What you are actually accomplishing in your life. | 1 | 2 | 3 | 4 | 5 |
| 7. | Your physical appearance - the way you look to others. | 1 | 2 | 3 | 4 | 5 |
| 8. | Your ability to adjust to change in your life. | 1 | 2 | 3 | 4 | 5 |
| 9. | Your life as a whole. | 1 | 2 | 3 | 4 | 5 |
| 10. | Overall contentment with your life. | 1 | 2 | 3 | 4 | 5 |
| 11. | The extent to which your life has been as you want it. | 1 | 2 | 3 | 4 | 5 |
| Specific comments: | | | | | | |

| **VI. Overall Impressions** | | | | |
| --- | --- | --- | --- | --- |
| **Please assess how each of the following have changed over the past 4 weeks compared to your typical state:** | | **Better** | **Same** | **Worse** |
| 1. | Overall my physical well-being is: | 1 | 2 | 3 |
| 2. | Overall my mental/emotional state is: | 1 | 2 | 3 |
| 3. | Overall my ability to handle stress is: | 1 | 2 | 3 |
| 4. | Overall my enjoyment of life is: | 1 | 2 | 3 |
| 5. | Overall my quality of life is: | 1 | 2 | 3 |
| Specific comments: | | | | |
